# Radiofrequency-Induced Intradiscal Nucleoplasty Chronic Low Back Pain Secondary To Lumbar Disc Herniation

**DOI:** 10.5704/MOJ.1307.009

**Published:** 2013-07

**Authors:** DW Lee, ESY Loh, CC Kueh, JH Poi, T Francis, KC Koh, NN Wazir, H Singh

**Affiliations:** Clinical School, International Medical University, Seremban, Malaysia; Clinical School, International Medical University, Seremban, Malaysia; Clinical School, International Medical University, Seremban, Malaysia; Clinical School, International Medical University, Seremban, Malaysia; Clinical School, International Medical University, Seremban, Malaysia; Clinical School, International Medical University, Seremban, Malaysia; Clinical School, International Medical University, Seremban, Malaysia; Clinical School, International Medical University, Seremban, Malaysia

## Abstract

We set out to assess the efficacy of radiofrequency-induced intradiscal nucleoplasty in reducing pain in symptomatic patients with MRI-defined lumbar disc herniation and their satisfaction with the procedure. We compared the patients’ pain intensity and severity of disability scores before and after undergoing the procedure in a retrospective questionnaire. These patients reported statistically significant reduction of pain intensity and disability level after the procedure. We conclude that radiofrequencyinduced intradiscal nucleoplasty is an acceptable alternative minimally invasive procedure in relieving the symptoms of patients with lumbar disc herniation.

**Key Words:**

Radiofrequency-induced intradiscal nucleoplasty, coblation therapy, percutaneous lumbar disc decompression, intervertebral disc herniation, low back pain

## Introduction

Chronic low back pain is a major social, economic, and healthcare issue. The studies evaluating chronic low back pain estimated that the average age-related prevalence of persistent low back pain as 12% in children and adolescents, 15% in adults, and 27% in the elderly^1^-^3^. Internal disc disruptions and disc herniations are common causes of low back and lower extremity pain which may become chronic, if left undiagnosed or untreated. These patients usually present with low grade continuous back pain, acute in onset resulting from lifting a heavy object, bending or an injury to the back. The pain often radiates to the hips and legs and may be associated with numbness and paraesthesia. It is frequently exacerbated by sitting up which increases pressure while lying down relieves stress on the disc space. In some severe cases, patients may develop sciatica, weakness of the lower limbs or cauda equina syndrome. Contained disc herniation can be confirmed by magneticresonance imaging (MRI) of the spine which reveals disc bulges or protrusions of the intervertebral disc of less than 5mm.

The mainstay of management in these patients is mostly symptomatic palliations which include long term or intermittent analgesics, physical therapy, behavioral management, and psychotherapy^4^. Minimally invasive approach in lumbar disc surgery has gained wide acceptance among investigators and patients alike; and is the first-line treatment recommended by insurers (Office of Clinical Standards and Quality, Health Care Financing Administration, Centre for Medicare/Medicaid Services 2006)^5^-^7^. Previous studies on radiofrequency technology in treating low back pain have demonstrated statistically significant reduction in pain scores with no worsening at 6 to 12 month follow-up, significant reduction in intradiscal pressure and better results compared to epidural steroid injections 8.

Radiofrequency intradiscal nucleoplasty (RIN) is a minimally invasive procedure for disc decompression that utilizes patented coblation technology for ablating and coagulating the intervertebral disc. This procedure uses a fraction of radiofrequency energy required by traditional radiofrequency energy systems in a process referred to as coblation. The technology creates a low-temperature (52°C) plasma field of ionized particles that breaks down organic molecular bonds within the tissue, creating small channels in the disc. The result is that a small portion of the nucleus pulposus is coagulated, reducing intradiscal pressure. Subsequently, pressure on the annulus fibrosus and nerve root is also reduced 9.

The proposed advantage of this technology is that the procedure provides for a controlled and highly localized ablation, resulting in minimal collateral damage to surrounding tissue. There is decreased morbidity and shorter recovery time associated with this procedure. RIN has become increasingly popular in the last three decades because of its good clinical success demonstrated in properly selected patients^9^.

## Materials and Methods

This study was undertaken in an orthopaedic clinic of a private hospital in Kuala Lumpur. Thirty-six patients with symptomatic low back pain and contained lumbar disc herniation confirmed with MRI who underwent RIN from year 2006 to 2008 were included in the study. All procedures were performed by a single surgeon. These patients responded in a retrospective questionnaire ( by post, phone interview and e-mails) evaluating their condition before and after treatment with RIN.

Two outcome measurement tools, namely, the Oswestry Low Back Pain Disability Questionnaire (OLBPDQ) and Visual Analogue Scale of Pain (VAS) were included in the questionnaire^10^,^11^. The OLBPDQ consists of 10 questions related to 10 different aspects of disability, which include pain intensity, personal care, lifting, walking, sitting, standing, sleeping, sex life, social life and travelling. Patients were asked to grade the extent of their functional disability experienced before and after treatment using a scale from A to F in the form of functional statements with each alphabet representing a score, i.e. A=0; B=1; C=2; D=3; E=4; F=5. Score 0 indicates highest function and 5 indicates lowest function. The sum of the scores from each question constitutes the Oswerstry Disability Index (ODI) ranged from a minimum score of 0 to a maximum of 50 11. Statistical analyses (paired T-test) comparing the means for each category in the OLBPDQ as well as the total score were then performed.

The patient’s pain score was evaluated using the VAS on a nominal scale of 0 (no pain) to 10 (worst pain ever) as experienced before and after treatment. The student paired Ttest was used for comparison of means. Statistical significance was defined as p < 0.05.

## Results

Thirty six patients participated in this study. Each of them indicated their pre- and post-treatment score for the 10 disability parameters in the Oswestry Low Back Pain Disability Questionnaire. The mean Oswestry Disability Index (ODI) for each disability parameter is shown in Table I.

Except for ‘personal care’, there was statistical significance between the mean ODI pre- and post-treatment (p < 0.05). Similarly, with regards to pain intensity, the patients completed the Visual Analogue Score for pre- and posttreatment. (See Table II) The difference between the mean VAS score pre- and post treatment was statistically significant (p < 0.05)

## Discussion

Our study showed that pain intensity, lifting, walking, sitting, standing, sleeping, sex life, social life and travelling significantly improved after treatment with radiofrequencyinduced intradiscal nucleoplasty as measured by the ODI and VAS. Our results demonstrated the efficacy of Intradiscal Nucleoplasty in improving symptoms and daily functioning of patients with disc herniation after treatment.

In our study, the mean ODI post-treatment was 9.80. A previous study found that the mean ODI of normal populations was 10.1^9^,^11^. A comparison of the ODI from our study to the normal population illustrates an improvement in quality of life of patients who had undergone the procedure to almost the level of normal populations.

There were statistically significant differences between pretreatment and post-treatment scores in every aspect measured by the ODI except in personal care (p>0.05). This may be due to the fact that the pre-treatment scores for personal care were not significantly higher compared with ideal levels.

Three patients in our study reported worst scores post treatment. It was noted that these three patients had expanded indications. They required more aggressive modality of therapy for their condition but they opted to undergo RIN instead, which would be suboptimal in addressing their problem. Hence, we infer that strict adherence to the stated indications for intradiscal nucleoplasty should be advised for desired outcome^12^.

This study has several limitations such as the relatively small sample size, possible recall bias as well as variability in the individual perception relating to pain intensity and disability level.

**Table I:**
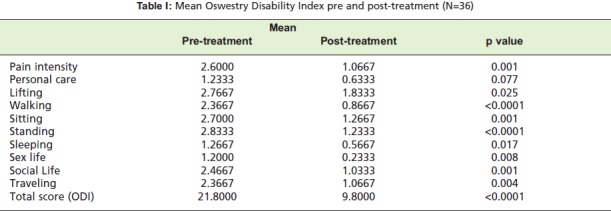
: Mean Oswestry Disability Index pre and post-treatment (N=36)

**Table II:**
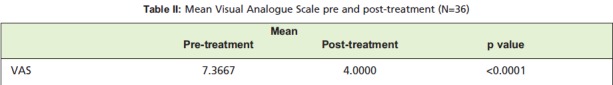
: Mean Visual Analogue Scale pre and post-treatment (N=36)

## Conclusion and Recommendation

Radiofrequency-induced intradiscal nucleoplasty is an acceptable minimally invasive procedure for contained lumbar disc herniation as it gives good pain relief and reduces disability. We recommend this procedure in carefully selected patients with chronic low back pain due to lumbar disc herniation.
